# 3D printed silicon-few layer graphene anode for advanced Li-ion batteries†

**DOI:** 10.1039/d1ra06643a

**Published:** 2021-10-29

**Authors:** Hossein Beydaghi, Sara Abouali, Sanjay B. Thorat, Antonio Esau Del Rio Castillo, Sebastiano Bellani, Simone Lauciello, Silvia Gentiluomo, Vittorio Pellegrini, Francesco Bonaccorso

**Affiliations:** Graphene Labs, Istituto Italiano di Tecnologia via Morego 30 16163 Genoa Italy; BeDimensional S.p.A Lungotorrente Secca 30R 16163 Genoa Italy f.bonaccorso@bedimensional.it

## Abstract

The printing of three-dimensional (3D) porous electrodes for Li-ion batteries is considered a key driver for the design and realization of advanced energy storage systems. While different 3D printing techniques offer great potential to design and develop 3D architectures, several factors need to be addressed to print 3D electrodes, maintaining an optimal trade-off between electrochemical and mechanical performances. Herein, we report the first demonstration of 3D printed Si-based electrodes fabricated using a simple and cost-effective fused deposition modelling (FDM) method, and implemented as anodes in Li-ion batteries. To fulfil the printability requirement while maximizing the electrochemical performance, the composition of the FDM filament has been engineered using polylactic acid as the host polymeric matrix, a mixture of carbon black-doped polypyrrole and wet-jet milling exfoliated few-layer graphene flakes as conductive additives, and Si nanoparticles as the active material. The creation of a continuous conductive network and the control of the structural properties at the nanoscale enabled the design and realization of flexible 3D printed anodes, reaching a specific capacity up to ∼345 mA h g^−1^ at the current density of 20 mA g^−1^, together with a capacity retention of 96% after 350 cycles. The obtained results are promising for the fabrication of flexible polymeric-based 3D energy storage devices to meet the challenges ahead for the design of next-generation electronic devices.

## Abstract 2

Novel 3D printed anodes based on Si and wet-jet milling-exfoliated few-layer graphene are produced by fused diffusion modelling (FDM) technique and used in Li-ion batteries.

## Graphical abstract

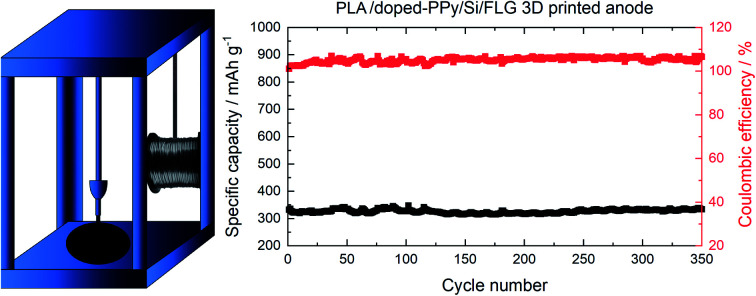

## Introduction

The application of Li-ion batteries has grown exponentially in recent decades due to the increasing demand for different emerging technologies, *e.g.*, portable electronics and plug-in hybrid electric vehicles.^1,2^ The increasing interest in Li-ion battery technology mainly relies on their high energy density (*e.g.*, 150–270 W h kg^−1^),^3^ low self-discharge (between 0.35% and 2.5% per month, depending on the state of charge),^4^ and stable cyclic performance.^5–7^ To fulfil the growing demand for advanced Li-ion batteries, great efforts have been dedicated to the development of new electrode materials capable to improve the energy capacities and lifetimes of the currently available technologies.^8,9^ Graphite, commonly used as the anode material in prototypical Li-ion batteries, has a limited theoretical capacity of 372 mA h g^−1^.^10^ Therefore, several alternatives to graphite have been widely investigated,^11–15^ among which silicon (Si) has been demonstrated to be an excellent candidate thanks to its extraordinary theoretical capacity of 4200 mA h g^−1^ (close to that of metallic Li) and low de-lithiation voltage (*i.e.*, ∼0.4 V *vs.* Li/Li^+^).^14,16–19^ In this context, numerous attempts exploited the combination of Si and carbon materials in hybrid electrodes,^14,18,20,21^ aiming to overcome the low electrical conductivity of Si,^22^ low initial coulombic efficiency,^23^ and the electrode instability caused by the Si swelling and contraction upon lithiation and de-lithiation, respectively.^23–25^

Besides exploring different candidate materials, the electrodes architecture design has become a research hot topic.^26^ In particular, the optimization of the electrode architecture can affect the transport of ions and electrons in the electrode,^27,28^ determining the final performance of the device. Recently, three-dimensional (3D) electrode architectures have been proposed to improve the ion transport process of the traditional electrodes, since their high electrochemically accessible surface area coupled with controlled porosity can be the key to unlock the full potential of the active materials.^29,30^ These features are particularly appealing for the realization of thick (>100 μm) electrodes, in which the underlying layers typically poorly contribute to the capacity of the electrode because of the intrinsic mass transport limits for Li ions in the electrolyte and the electrical resistance for electrons in the solid phase of the electrode.^29,31^ The possibility to fully exploit the active materials in thick electrodes can prospectively reduce the cell manufacturing costs while improving the energy density of the whole packed devices,^31,32^ as well as retaining optimal rate capabilities.

Driven by emerging technologies that use Li-ion batteries with customized form factors, 3D printing techniques have gained particular attention as effective paths to make complex architectures with controlled geometries and sizes.^33–35^ The first 3D-printed Li-ion battery was realized using an extrusion-type 3D printer, in which the printable inks were composed of LiFePO_4_ and Li_4_Ti_5_O_12_ active materials.^36^ In the 3D printing techniques area, the fused diffusion modelling (FDM) is a low-cost, simple, and high-throughput technique for printing polymeric products. For the case of FDM-printed electrodes, the electrode thickness can be controlled by adjusting the number of printed layers, the diameter of the printing nozzle, and the printing speed.^37^ Although the electrodes prepared by other 3D printing techniques, *i.e.*, direct ink writing, can deliver high energy densities, (*e.g.*, 69.41 J cm^−2^ at ∼2.99 mW cm^−2^),^38^ the viscosity of the ink and the need for post-treatment processes of the electrodes, such as freeze-drying and thermal annealing, may negatively affect the manufacturing throughput.^26^

The use of FDM to print 3D polymer-based electrodes for Li-ion batteries have been demonstrated using commercial graphene/polylactic acid (PLA) filament.^39^ However, the low mass ratio of the active material (*e.g.*, 8 wt%) in the polymeric matrix, limited the discharge capacity of anode (calculated on the mass of the active material) to 15.8 mA h g^−1^ at the current density of 10 mA g^−1^. The electrochemical performances were significantly lower compared to the one achieved by conventional Li-ion battery anodes.^40^ Similarly, a FDM-printed Li-ion battery has been produced using polymer filaments prepared by mixing the cathode (lithium manganese oxide) and anode Li_4_Ti_5_O_12_ active materials with the electrically conductive ones (*i.e.*, carbon black, graphene, multi-walled carbon nanotubes) blended with PLA.^40^ The 3D printed anode exhibited a discharge capacity of 3.5 mA h cm^−3^ at the current density of 20 mA g^−1^, which was used to power electronic devices, such as liquid crystal display sunglasses and light-emitting diodes. More recently, 3D printed anodes made of graphite/propylene carbonate and poly(ethylene glycol) dimethyl ether as plasticizers, and carbon black and carbon fibre as conductive materials, have shown a discharge capacity of 140 mA h g^−1^ at the current density of 37.3 mA g^−1^, indicating a substantial progress of the FDM-printed battery performances compared to the previous 3D printed technologies.^26^

Despite these advancements, a major limitation for 3D electrodes printed employing the FDM technique is the intrinsic low electrical conductivity (*i.e.*, from 10^−9^ to 10^−14^ S cm^−1^) of the polymer used in the filament.^40^ To overcome this problem, the conventional polymers for FDM 3D printing are commonly blended with electrically conducting polymers such as polypyrrole (PPy). However, pristine PPy has an electrical conductivity of ∼10^−6^ S cm^−1^ and is typically doped with carbon black to exhibit electrical conductivities in the 0.8–40 S cm^−1^ range,^41–43^ which is acceptable for the formulation of Li-ion battery electrodes.^44–46^ Noteworthy, Si-PPy composites have also been synthesized by coating the Si particles with PPy, thus improving both the electrical conductivity and the electrochemical stability of the electrodes upon charge/discharge cycles.^47,48^

In this work, it is shown an electrically conductive filament for FDM printing based on PLA/carbon black-doped PPy blend combined with Si nanoparticles and wet-jet milling-exfoliated few-layers graphene (WJM-FLG) as the active material and electrically conductive filler, respectively. By considering that there is not any previous study on the fabrication of a Si-based anode by means of FDM method, we opted to investigate the feasibility of such 3D printing technique using a simple disk-shape architecture, focusing our attention on the formulation of printable filament, as well as the consequent electrode performance optimization. By engineering the doping of PPy with carbon black, the printable filament achieves an electrical conductivity as high as 5.19 S cm^−1^, which is 9 order of magnitude higher than conductivity of the bare PLA filament, and only one order of magnitude superior to the conductivity reported for conductive filaments produced through FDM 3D printing (*e.g.*, 0.4 S cm^−1^).^26^ The distinctive electrical properties reached by our filament allow us to print 3D flexible polymeric anodes for Li-ion batteries. As assessed for both pristine graphene and its derivatives (*e.g.*, functionalized reduced graphene oxide),^49–55^ the WJM-FLG create inter-layered structures that provide transport channels for electrons and ions, improving the electrical and ionic (Li^+^) conductivity compared to the reference (*i.e.*, WJM-FLG-free) electrodes.^56^ Moreover, both WJM-FLG and doped PPy uniformly coat the surface of the Si nanoparticles, limiting the volumetric expansion of electrodes during Si lithiation.^19,57^ Meanwhile, the conductive WJM-FLG/doped PPy network effectively surrounds the Si nanoparticles to prevent the volume change upon the de-lithiation, avoiding aggregation effects that degrade the anode performances. The optimized 3D printed electrode shows a specific capacity up to 345 mA h g^−1^ at the current density of 20 mA g^−1^ with a capacity retention of 96% after 350 cycles. Our results prove the possibility to specifically use the FDM method as low-cost and high-speed 3D printing technique, simplifying the scaling-up of the electrode manufacturing compared to other 3D printing technologies.

## Result and discussion

### Filament and electrode characterization

**Fig. 1:**
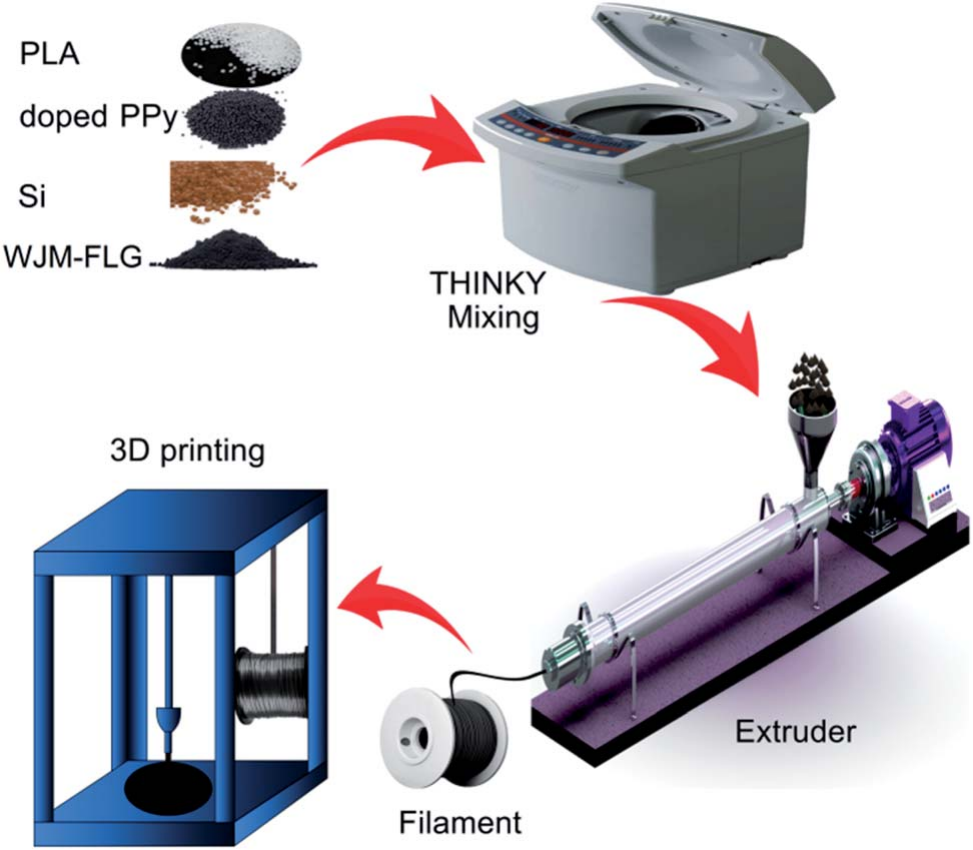
Schematic illustration of the 3D printing of the Li-ion battery anodes.

A schematic illustration of the electrode fabrication process is sketched in Fig. 1, displaying (i) the filament preparation and (ii) the 3D printing process. The compositions of the formulated filaments are summarized in Table 1, along with the corresponding measured electrical conductivities. Three types of filaments were produced, *i.e.*: PLA, PLA/carbon black doped PPy, and PLA/carbon black-doped PPy/Si/WJM-FLG (hereafter named FP, FPP and F*x*, respectively, in which *x* indicates a specific formulation used for PLA/doped PPy/Si/WJM-FLG filament, as defined in Table 1). The investigated filament compositions were chosen based on the feedback provided by the electrical and mechanical characterizations of the filaments, as well as the electrochemical data of the corresponding electrodes (as discussed later in the text). All the filaments were produced by extrusion process and directly used for the FDM 3D printing (see details in Experimental section). Noteworthy, the application of PLA-based filaments for FDM 3D printing has been reported in previous works, pointing out its printability and biodegradable properties.^58^ However, the electrical conductivity of the pure PLA (*i.e.*, FP sample) is 3.25 × 10^−9^ S cm^−1^, which is inadequate for the development of electrode for Li-ion batteries.^59^ By doping the PLA with 10 wt% of carbon black-doped PPy, the electrical conductivity of the pristine PLA is improved by ∼5 orders of magnitude. The addition of WJM-FLG into the mixture further increases the electrical conductivity of the samples, up to a maximum value of 5.19 S cm^−1^ for sample F5. It is worth pointing out that the filaments with PLA contents lower than 45 wt% were not FDM printable due to their brittleness.

**Table 1:** Composition and electrical conductivity of the investigated FDM filaments

Sample	Composition (wt%)				Conductivity
	PLA	Doped-PPy	Si	WJM-FLG	*σ* (S cm^−1^)
FP	100	0	0	0	3.25 × 10^−9^
FPP	90	10	0	0	7.69 × 10^−4^
F1	80	7	11	2	2.77 × 10^−2^
F2	70	11	16	3	5.73 × 10^−1^
F21	70	11	15	4	7.46 × 10^−1^
F22	70	11	15.5	3.5	7.12 × 10^−1^
F23	70	11	16.5	2.5	5.24 × 10^−1^
F24	70	10	16	4	6.12 × 10^−1^
F25	70	10.5	16	3.5	5.88 × 10^−1^
F26	70	11.5	16	2.5	5.36 × 10^−1^
F3	60	15	21	4	2.16
F4	50	18	27	5	4.78
F5	45	20	29.5	5.5	5.19

The X-ray diffraction (XRD) patterns of the pristine Si and the F5 powders are shown in Fig. S4.† The peak located at a diffraction angle (2*θ*) of 26.65° corresponds to the (002) reflection of the WJM-FLG flakes,^60,61^ while the sharp peaks located at 28.42, 47.27, 55.94, 69.01 and 76.26° correspond to the (111), (220), (311), (400) and (331) reflections of Si nanoparticles, respectively, matching with the characteristic peaks of cubic (*Fd*3̄*m*) Si.^62,63^ Besides, the peak centred at 16.57° is assigned to PLA, in agreement with previous studies.^64^

**Fig. 2:**
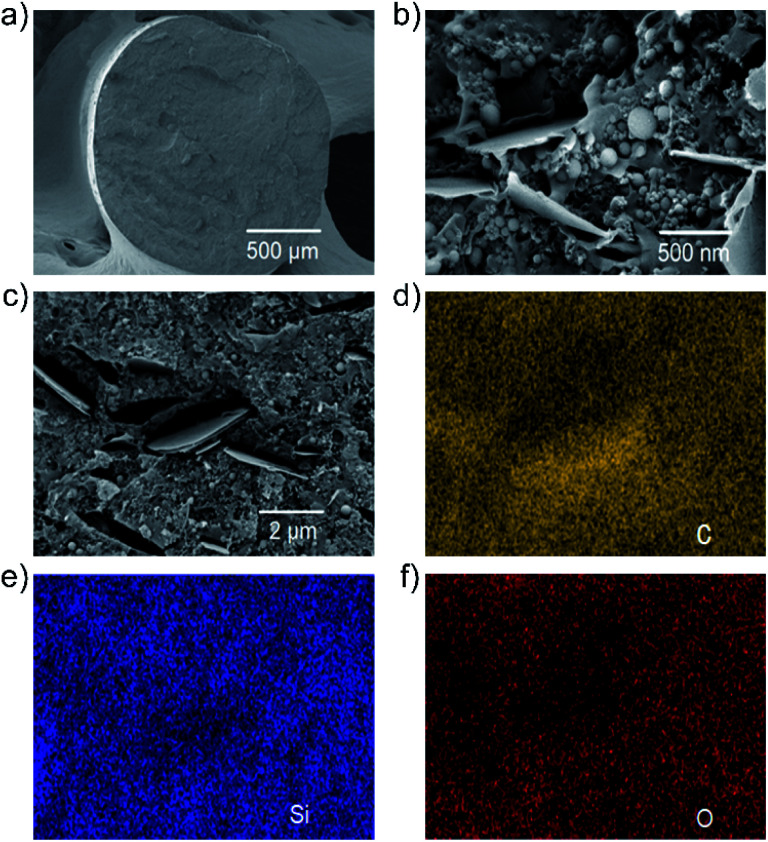
(a and b) Cross sectional HR-SEM images of the F5 filament at increasing magnifications. (c) Back-scattered and secondary electrons image of cross-sectional F5 filament. EDX map for (d) C (M line at 0.28 keV) (e) Si (M line at 1.78 keV) and (f) O (M line at 0.54 keV).

The morphology of the filaments was evaluated by high-resolution scanning electron microscopy (HR-SEM). Fig. 2a and b show the cross-sectional HR-SEM images of the F5 filament at increasing magnifications. In low magnification HR-SEM image (Fig. 2a), the produced filament shows a homogeneous compact structure without the presence of structural defects, *e.g.*, cracks. By increasing the magnification, HR-SEM imaging (Fig. 2b) shows the presence of Si nanoparticles and WJM-FLG flakes, which are both randomly distributed within the polymeric matrix. The back-scattered and secondary electron HR-SEM image of the F5 filament cross-section (Fig. 2c) reveals that voids are introduced in the proximity of the WJM-FLG flakes. As observed in literature,^18,51^ such nanoscale pores can play a major role in containing the volumetric expansion of Si-based anodes during the lithiation processes, while the 2D morphology of the WJM-FLG flakes is effective to preserve the electrical connection of Si nanoparticle as they contract upon the de-lithiation process. Fig. 2d shows the EDX maps for C, Si, and O corresponding to the HR-SEM image reported in Fig. 2c. These data indicate a homogeneous dispersion of the active materials within the polymeric matrix.

### Electrochemical characterization

**Fig. 3:**
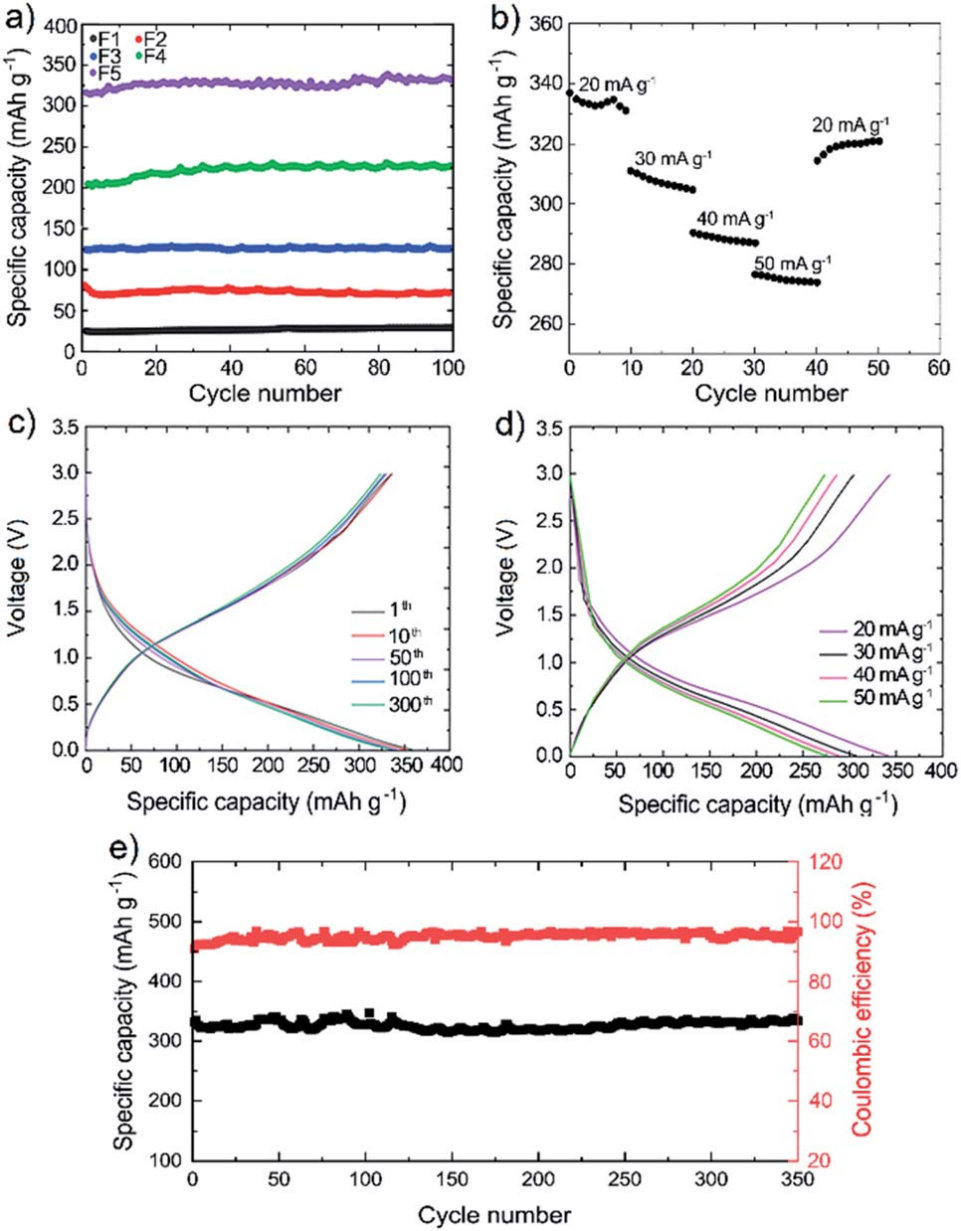
(a) Specific capacity of the 3D printed electrodes, as calculated by galvanostatic charge/discharge curves at the current density of 20 mA g^−1^. (b) Rate capability of the 3D printed F5 electrode tested at different current densities ranging from 20 mA g^−1^ to 50 mA g^−1^. (c) Charge/discharge curves of the 3D printed F5 electrode at representative cycles (1st, 10th, 50th, 100th and 300th cycles) at the current density of 20 mA g^−1^. (d) Charge/discharge curves of the 3D printed F5 electrode at different current densities ranging from 20 mA g^−1^ to 50 mA g^−1^. (e) Long-term cyclic performance and coulombic efficiency of the half-cell assembled with the 3D printed F5 electrode at the current rate of 20 mA g^−1^.

Fig. 3 and S7† show the electrochemical performance of the 3D printed electrodes in half-cell configuration. The practical capacity that can be achieved by the electrode depends on the mass ratio of the active material (Si), as well as by other parameters, including the electrical conductivity and the porosity that can be tuned by changing the WJM-FLG or carbon black-doped PPy contents. Due to the complexity of the structure, the relationship between capacity and these parameters may be non-linear, indicating the need of experimental tests for the optimization of the electrode formulation. Therefore, various electrodes have been prepared by varying the content of the active material (areal mass loading of Si between 1.6 to 3.8 mg cm^−2^), PLA and carbonaceous conductive fillers. By fixing the content of PLA and carbon black-doped PPy to 70 and 11 wt%, respectively, the F2 electrode, composed by 16 wt% of Si and 3 wt% of WJM-FLG, shows the highest specific capacity of ∼70 mA h g^−1^ at the current density of 20 mA g^−1^ (Fig. S7a and c†). By increasing further the Si : WJM-FLG weight ratio, the specific capacity of the 3D printed electrode decreases because of the low conductivity of the composing filament, *i.e.*, 0.524 S cm^−1^ (see Table 1). Noteworthy, an excessive content of WJM-FLG (low Si : WJM-FLG weight ratio, *i.e.*, F21 electrode) can negatively affect the electrolyte accessibility to the active materials, thus decreasing the specific capacity compared to the optimal case (F2 electrode). By fixing the weight content of PLA and Si at 70 and 16 wt%, respectively, and varying the weight content of carbon black-doped PPy and WJM-FLG, the F2 electrode still shows the highest specific capacity, as shown in Fig. S7b and d.† Although both carbon black-doped PPy and WJM-FLG are conductive materials, they provide different pathways for electron/ion transport during charge/discharge cycles. Due to their 2D morphology, the WJM-FLG flakes provide a long-range connected network within the filament structure (Fig. 2c). Meanwhile, carbon black-doped PPy tends to coat the surface of Si nanoparticles, thus creating a conductive layer, ensuring the electrical contact between the Si nanoparticles and the surrounded matrix.^45^ Based on the above discussion and experimental data, the optimal weight composition of carbon black-doped PPy : Si : WJM-FLG composite has been found to be (3.7 : 5.3 : 1), which corresponds to the F2 filament.

More in detail, Fig. 3a shows the specific capacity of representative 3D printed electrodes, as calculated by galvanostatic charge/discharge curves at the current density of 20 mA g^−1^. By increasing the weight percentage of Si from 11 wt% in F1 to 29.5 wt% in F5, the specific capacity of the electrode increases from 25 mA h g^−1^ to 334 mA h g^−1^ since more active material participates in the lithiation/delithiation processes. Meanwhile, our conductive additives, *i.e.*, carbon black-doped PPy and WJM-FLG, are considered in the electrode formulation to electrically connect the active Si.^18^ Actually, the full exploitation of the theoretical capacity of Si (*i.e.*, 4200 mA h g^−1^) still represents a primary challenge for 3D printed Si-based Li-ion batteries, in which high binder content is typically needed compared to conventional batteries.^65–67^ Noteworthy, even in F5, the PLA content is still as high as 45 wt% to guarantee adequate mechanical properties needed for the electrode printability, while guaranteeing a maximum electrical conductivity of 5.19 S cm^−1^. All the prepared electrodes with various formulations, from F1 to F5, show a stable cyclic performance, which is attributed to the uniform dispersion of Si nanoparticles within the filament, as well as to the flexibility and robustness of the electrode architecture.^19^ Indeed, these electrochemical data, together with our microscopy characterization results, confirm that: (1) the uniform dispersion of Si nanoparticles, (2) the Si nanoparticles connection through conductive pathways introduced by WJM-FLG flakes and carbon black-doped PPy and (3) the voids introduced nearby the WJM-FLG can hinder the degradation caused by the volume changes and reaggregation of Si nanoparticles during the lithiation/de-lithiation processes.^21,68^

Fig. 3b shows the rate capability of the optimized 3D printed electrode (F5). The slight increase of the rate performance after the first 5 cycles at 20 mA g^−1^ may be attributed to the gradual activation of Si nanoparticles at low current rate, as also reported in previous works.^69,70^ By increasing the current density from 20 mA g^−1^ to 30, 40 and 50 mA g^−1^, the specific capacity decreases from 334 to 308, 288 and 275 mA h g^−1^, respectively. Nevertheless, the F5 anode still preserves 82.3% of its initial specific capacity at the current density of 50 mA g^−1^. Once the current density is decreased again to the 20 mA g^−1^, the electrode recovers more than 95% of its initial capacity.^1^ The optimal rate performance of the 3D printed F5 anode is attributed to the conductive network created by the carbon black-doped PPy and WJM-FLG flakes, as well as the buffering of the voids in the 3D electrode structure that improve the Li^+^ accessibility to the active surface area^19,71,72^ while containing the volumetric changes during charge/discharge cycles.^19,21,68^Fig. 3c shows the charge/discharge curves of the F5 electrode for representative cycles at the current density of 20 mA g^−1^. The overlap of the charge/discharge profiles from the 1^st^ to 300^th^ cycle (Fig. 3c) confirms the reversibility of the electrochemical reactions and stability of the electrode over cycling. Fig. 3d shows the charge/discharge profiles of the F5 electrode at different current densities from 20 to 50 mA g^−1^. The overall shape of the curves is unaltered upon the increase of the current density, while the specific capacity decreases gradually due to kinetic limits of the electrochemical reactions, which is consistent with the results shown in Fig. 3b.

Fig. 3e shows the specific capacity and coulombic efficiency of the 3D printed F5 electrode over 350 cycles at 20 mA g^−1^. The electrode progressively increases its coulombic efficiency from 90% in the first cycle up to 96% after 10 cycles. Even if marginal, the irreversible capacity is attributed to the solid-electrolyte interphase (SEI) formation at the electrode/electrolyte interface, as well as to the irreversible insertion of Li^+^ into Si nanoparticles.^18,73^ After the first 10 cycles, the anode preserves its coulombic efficiency, showing stability over 350 cycles. In fact, at the 350^th^ cycles, the specific capacity is 327 mA h g^−1^, which corresponds to a capacity retention as high as 95%. The porous structure of the electrode, introduced by a 3D printing technique, boosts the electron/ion transport within the electrode structure, giving rise to the outstanding long-term cycling stability of the produced electrodes achieved in this work.^74–76^ Overall, our data prove an optimal cyclic performance of the 3D printed Si-based electrodes incorporating carbonaceous conductive components, in agreement with previous works.^77^

**Fig. 4:**
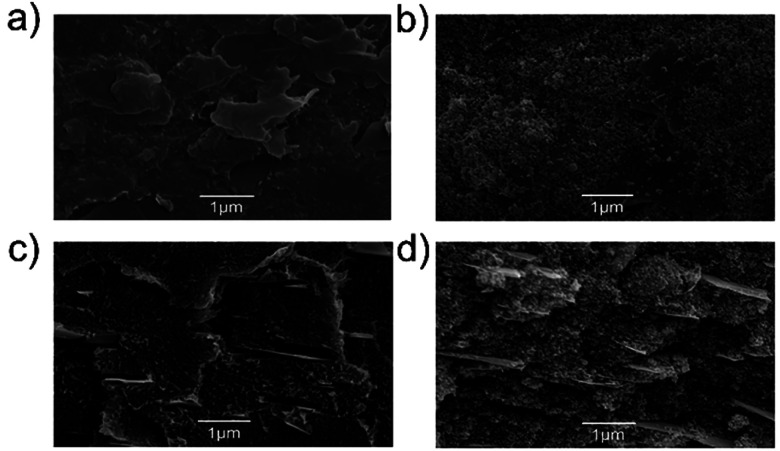
(a) Top-view HR-SEM image of the as-prepared 3D printed F5 electrodes and (b) after 350 galvanostatic charge/discharge cycles at the current density of 20 mA g^−1^. (c) Cross-sectional HR-SEM image of the fresh F5 electrode and (d) after 350 galvanostatic charge/discharge cycles at the current density of 20 mA g^−1^.

The 3D structure of the electrodes, along with their flexibility (Fig. S6a†), plays an important role in maintaining their cyclic stability during the Li charge/discharge process.^19^ In particular, the flexibility of the electrode can help to ensure the mechanical integrity of the electrode structure during volume changes arising from Si lithiation/de-lithiation processes.^19^Fig. 4 shows the top-view and cross-sectional HR-SEM images of the 3D printed F5 electrode before and after 350 cycles of charge/discharge.

The HR-SEM images show that the electrode has preserved its main structure after 350 cycles, and the observed particles on the surface of the cycled electrodes are attributed to the formation of the SEI layer. The WJM-FLG flakes are distributed between the polymeric matrix, forming a conducting porous framework, and creating voids (*i.e.*, pores) similarly to the case of the corresponding FDM filaments. The porosity created nearby the WJM-FLG flakes facilitates the access of the electrolyte for rapid intercalation of Li^+^ into the Si nanoparticles, while containing the volumetric expansion of the electrode during the lithiation process.^21,78^ During the de-lithiation process, the volume of the Si nanoparticles contracts, but the conductive network given by WJM-FLG flakes and carbon black-doped PPy maintains the electrical connection of the active nanoparticles. Furthermore, the encapsulation of Si nanoparticles within the PLA filament prevents the detachment of Si nanoparticles from the 3D printed anode (see Fig. S8†).^21,79^ Lastly, the thickness of F5 electrode before and after 350 cycles is 200 and 220 μm, respectively. These values confirm the positive effect of the presence of voids, determined by the 3D printing process, limiting the Si volume expansion.

**Fig. 5:**
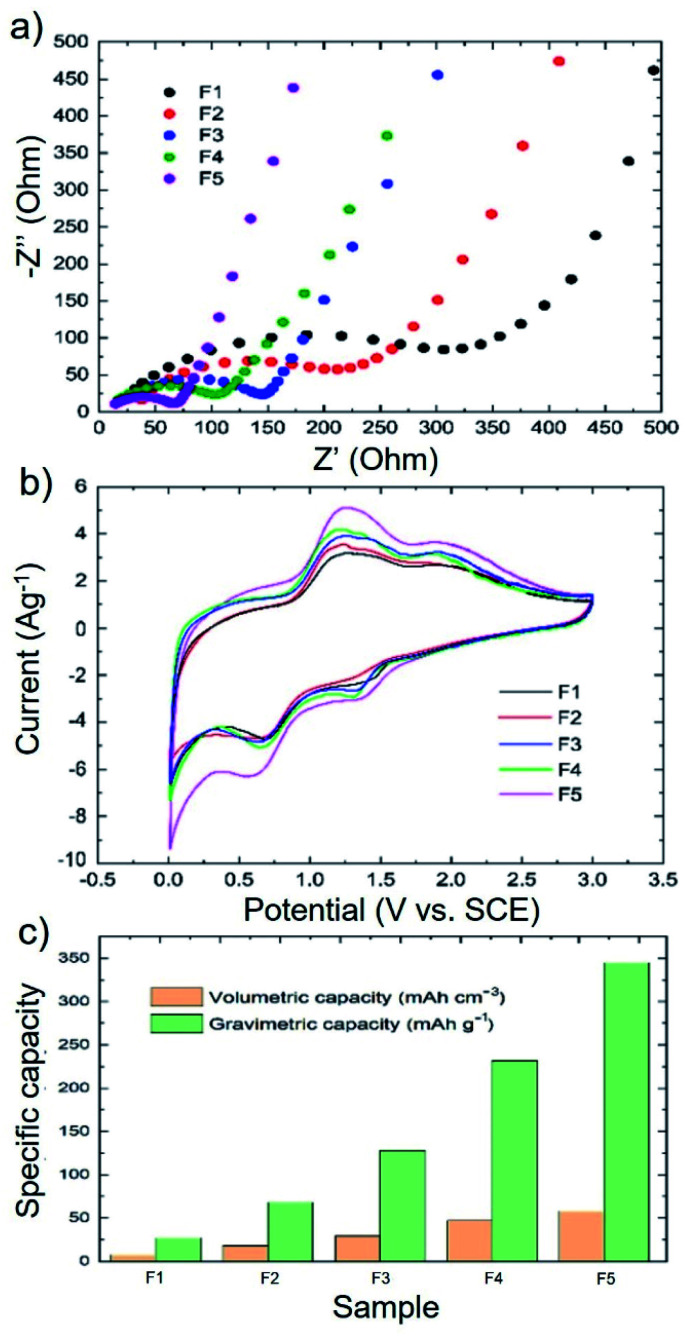
(a) Nyquist plots of the electrochemical impedance spectra of the 3D printed electrodes in the 0.01–200 kHz frequency range. (b) First cycle CV profile of the 3D printed electrodes at the scan rate of 0.1 mV s^−1^ in the potential range of 0–3 V. (c) Gravimetric and volumetric capacities of the investigated 3D printed electrodes.

The electron/ion transport behaviour of the 3D printed electrodes was evaluated through electrochemical impedance spectroscopy (EIS) measurements. Fig. 5a shows the Nyquist plots measured for F1–F5 electrodes, indicating that the charge transfer resistance (*R*_ct_) decreases from 295 Ω in F1 to 54 Ω in F5, being the PLA loading reduced from 85 wt% to 45 wt% in the mentioned electrodes. These results demonstrate the improvement of the electrical connection and charge transport between the Si and WJM-FLG flakes and carbon black-doped PPy with increasing the amount of these materials in the formulation of the 3D printed electrodes.^24,80^ Overall, the EIS results indicate that the cyclic stability and high-rate capabilities of the printed electrodes strongly depend on the formation of a 3D conductive WJM-FLG and carbon black-doped PPy-based network that confines the Si nanoparticles.^18,81^ Therefore, our electrode has been optimized by increasing the WJM-FLG weight percentage, until the threshold value for which the filaments were no longer printable.

To provide further insight on the electrochemical behaviour of 3D printed Si-based electrodes compared to conventional ones, Fig. 5b shows the cyclic voltammetry (CV) curves of our representative 3D printed electrodes measured at a scan rate of 0.1 mV s^−1^ from 0 to 3 V. Although two pairs of lithiation/de-lithiation peaks can be observed in cathodic/anodic scans, the location of the peaks has been shifted to higher potentials compared to the previous reports for Si-based electrodes.^1,81^ This observation is consistent with the charge/discharge curves presented in Fig. 3c. We speculate that the high amount (≥45 wt%) of a non-conductive polymer (*i.e.*, PLA) in the electrode structure can shift the electrochemical reactions to a higher voltage because of the high electrical resistance (*R*_ct_: 54–295 Ω). In the case of the 3D printed F5 electrode, the two reduction peaks located at 1.4 and 0.7 V correspond to the alloying of Si with Li, as well as the formation of the SEI layer.^82,83^ During the charging process, two oxidation peaks appear at 1.4 and 2.0 V, which are ascribed to the Li-ion extraction from the composite (de-lithiation). By comparing the different electrodes, the oxidation/reduction peaks tend to be more pronounced moving from F1 to F5 (+64% current increase). This means that the lithiation/de-lithiation processes are more efficiently carried out with decreasing the PLA content. Nevertheless, as previously discussed, the amount of WJM-FLG and PPy must be optimized to provide the optimal trade-off between electrochemical performance and printability.^84^

Fig. 5c compares the gravimetric (calculated on the mass of the active material) and volumetric capacities of the as-produced 3D printed electrodes. Our 3D printed anodes present capacities that significantly exceed those reported in relevant literature by other 3D printed anodes based on FDM 3D printing for Li-ion batteries (Table S1†) and tested under comparable experimental conditions.^26,39,40^ For example, Maurel *et al.*,^26^ reported graphite–PLA composite-based anodes with reversible capacity values around 200 mA h g^−1^ at the current density of 18.6 mA g^−1^. Reyes *et al.*,^40^ reported Li_4_Ti_5_O_12_-based anodes showing volumetric capacities around 3.5 mA h cm^−3^ at a current density of 20 mA g^−1^. Foster *et al.*,^39^ prepared the 3D printed anode from a commercial PLA/graphene-based filament, achieving a discharge capacity up to 15 mA h g^−1^ at a current density of 10 mA g^−1^.

It should be noted that our optimized 3D printed anode shows a specific capacity (345 mA g^−1^ relatively to mass of active materials and 101 mA g^−1^ relatively to entire mass of composite) ∼12 times lower than the Si theoretical capacity (4200 mA h g^−1^), thus there is plenty of room for further improvement in the anode formulation. At the current stage, it is still challenging to minimize the polymer wt% while maintaining the filament printability. Nevertheless, the use of the WJM-FLG flakes additive represents an optimal solution to create highly conductive filaments, thus solving the current limitations of the FDM 3D printed electrodes for Li-ion batteries. Our results may be used to create hierarchical electrode configurations combining macro-texturized architectures (*e.g.*, mesh-like structures)^85^ with our electrode microporosity to further boost the performances of the current 3D printed batteries.

## Experimental

### Materials

Ingeo Biopolymer 4043D PLA pellets were supplied from NatureWorks, LLC. Carbon black-doped PPy (conductivity: 30 S cm^−1^) was purchased from Merck. Si nanoparticles (100 nm) were supplied from Merck. The FLG powder was produced by exfoliating graphite with the WJM method^11,13,86–91^ followed by freeze-drying,^87,88^ as described in patent No. WO2017089987A1. Experimentally, a mixture of 200 g of graphite flakes (+100 mesh, Merck) and 20 L of *N*-methyl-2-pyrrolidone (>97%, Merck) was placed in the container and mixed by the mechanical stirrer (Eurostar digital Ika-Werke). The obtained mixture was pushed by a hydraulic piston into the WJM processor using a pressure ranging from 140 to 250 MPa. The mixture of graphite and NMP was forced to pass through a nozzle with a diameter of 0.3 mm. During this process, the shear forces and the cavitation, originated by the turbulence of the solvent during its passage through the nozzle, cause the exfoliation of the graphite into FLG. The obtained dispersion was cooled down by the chiller and transferred to another container. The as-produced dispersion was re-processed by the WJM system three times, using nozzles with different diameters of 0.2, 0.15 and 0.1 mm, respectively. The as-prepared FLG dispersion was dried using a rotary evaporator (Heidolph Heivap Industrial, FKV Srl, Italy) at a bath temperature of 80 °C. While the pressure decreased down to 5 mbar, 6 L of dimethyl sulfoxide were added (Merck KGaA, Germany). To obtain the FLG flakes powder (sample named WJM-FLG), the obtained mixture was kept in a refrigerator for 3 h at −15 °C, followed by freeze-drying for 50 h at a temperature of −80 °C and a pressure of 0.1 mbar. The details of the morphological, chemical and structural characterizations of the WJM-FLG are reported in the ESI (Fig. S1–S3†).

### Preparation of the 3D printable filament

Firstly, PLA pellets were powdered using the Ultra Centrifugal Mill (Retsch, ZM200, Germany) machine and dried at 85 °C overnight. The PLA, the carbon black-doped PPy powder, the Si nanoparticles and the WJM-FLG powder were mixed according to the formulation shown in Table 1, using a planetary mixer (Thinky, ARE 200, USA). A twin-screw extruder (Bandera, 45L/D, Italy) was used to produce the 3D printing filaments with a diameter of ∼1.75 mm. The extruder was cleaned with pristine PLA pellets and subsequently loaded with ∼50 g of the composite mixture. The temperature of the extruder was set between 175 and 205 °C. After the extrusion process, the as-prepared filament was rolled around a spool and stored in sealed plastic bags to keep it dry.

### 3D printing process

A 3D model of the anode electrode was designed *via* Solid works software. A disc electrode with a diameter of 1.0 cm and a thickness of 0.2 mm was designed and converted to Standard Triangle Language (STL) format. Then, the as-designed disc electrode was sliced using Cura software (Ultimaker). The as-prepared filament was used to print 3D electrodes using an IRA3D FDM 3D printer. The MK10 nozzle with input and output diameters of 1.75 and 0.4 mm, respectively, was used for the printing process. The temperatures of the nozzle and bed were set to 210 °C and 60 °C, respectively, to improve the adherence of the first printed layer. The printing speed was adjusted at 40 mm s^−1^ and the infill density was 100%. The details of the structural characterization of the as-produced filament are reported in the ESI (Fig. S4–S7†).

### Characterization

Transmission electron microscopy (TEM) measurements were carried out using a JEM 1011 (JEOL) transmission electron microscope (thermionic W filament), operating at 100 kV. The samples were prepared by depositing the 1 : 50 diluted WJM-FLG dispersion onto an ultrathin C-film on holey carbon 400 mesh Cu grids (Ted Pella Inc.). ImageJ software (NIH) was used to perform the statistical analysis of the lateral dimension of the WJM-FLG flakes. The surface morphology of the electrodes and cross-sectional morphology of the filaments were studied using a JEOL JSM-6490LA SEM Analytical SEM. Before the SEM imaging, the samples were coated with Au. The element mapping of the electrodes was accomplished using energy-dispersive X-ray spectroscopy (EDX)-coupled SEM, operating at 5 kV acceleration voltage. For the cross-sectional SEM imaging, the filament and electrode samples were carefully cut after immersion in liquid nitrogen and fixed in 90° tilted sample holder. XRD measurements were performed using a PANalytical Empyrean X-ray diffractometer using Cu Kα radiation from 10° to 80°. The Raman spectra of the samples were recorded using a micro-Raman spectrometer (Renishaw Invia 1000) with an excitation wavelength of 532 nm. For the XRD and Raman measurements, the samples were prepared by drop-casting the WJM-FLG-based dispersions onto Si/SiO_2_ substrates and dried under vacuum overnight. The thermal stability of the as-produced filaments was investigated by thermogravimetric analysis (TGA) using TGA Q500 (TA Instruments, USA). The TGA measurements were carried out in nitrogen atmosphere in the 50–800 °C temperature range, using a heating rate of 10 °C min^−1^. The tensile tests of the filaments were carried out using the Instron Dual Column Tabletop Universal Testing System 3365, with a jaw speed of 3 mm min^−1^. The electrical conductivity measurements were performed using a Loresta-GX MCP-T700 (Mitsubishi Chemical Analytech.).

The 2032 coin cells were assembled in an Ar-filled glovebox using the 3D printed discs as the free-standing electrodes and metallic Li disks as the counter electrode. The electrolyte consisted of LP30 (Solvionic, 1 M LiPF6 in dimethyl carbonate, DMC : ethylene carbonate, EC 1 : 1 v/v) embedded in a Whatman borosilicate separator. The coin cells were tested on a Biologic battery tester in a potential window between 0.01 and 3 V and at current densities ranging from 20 to 50 mA g^−1^. The CV measurements were performed with a scan rate of 0.1 mV s^−1^. The EIS data were acquired with an AC voltage amplitude of 0.02 V over a frequency range from 0.01 Hz to 200 kHz. The specific capacities were calculated over the mass loading of Si.

## Conclusions

In the present work, we presented the formulation of a novel conductive filament suitable for fused deposition modelling (FDM) 3D printing of anodes for Li-ion batteries. The filament is based on polylactic acid (PLA), carbon black-doped polypyrrole (PPy), Si and wet-jet milled produced few-layers graphene (WJM-FLG) flakes. The Si and WJM-FLG flakes act as the active and conductive materials, respectively, and their weight percentages were increased as much as possible to improve the electrochemical performances, while maintaining 3D printability. In the optimized filament structure, the Si nanoparticles are dispersed within carbon black-doped PPy and WJM-FLG flakes to avoid volumetric expansion and consequent material loss from the electrode. The optimized 3D printed anode shows gravimetric capacity up to 345 mA h g^−1^ and the volumetric capacity of 58.6 mA h cm^−3^ at the current density of 20 mA g^−1^. These values are the highest among those reported in literature for FDM 3D printed anodes.^26,39,40^ The as-produced 3D printed anode exhibit excellent cyclic stability and rate performance resulting from the 3D conductive carbonaceous framework, the porous network created by 3D printing, and the flexibility of the electrode. Although the specific capacities reached by our 3D printed electrode are still lower than those of conventional Si-based electrodes, the FDM 3D printing technology has the potential to simplify the fabrication of Li-ion battery components, while providing a unique solution for the realization of efficient thick (>100 μm) electrodes with a high energy density of the whole battery stack. Overall, our FDM 3D printed anodes shows promising flexibility and electrical conductivity, which may provide novel design for the manufacturing of portable electronics and energy storage devices, including Li–sulphur batteries and supercapacitors.

## Author contributions

Hossein Beydaghi: conceptualization, investigation, methodology, writing manuscript. Sara Abouali: conceptualization, investigation, methodology. Sanjay B. Thorat: filament preparation. Antonio Esau Del Rio Castillo: FLG synthesis and characterization. Sebastiano Bellani: conceptualization, writing manuscript. Simone Lauciello: material characterization. Silvia Gentiluomo: 3D printing. Vittorio Pellegrini: supervision, writing – review & editing, resources. Francesco Bonaccorso: supervision, writing – review & editing, resources.

## Conflicts of interest

There are no conflicts to declare.

## Supplementary Material

RA-011-D1RA06643A-s001
