# Supplementary material for: 3D printed silicon-few layer graphene anode for advanced Li-ion batteries
Source: RSC Adv. 2021 Oct 29;11(56):35051–60. doi: 10.1039/d1ra06643a (PMC9042803; doi:10.1039/d1ra06643a)
Supplement: RA-011-D1RA06643A-s001 [file RA-011-D1RA06643A-s001.pdf]

Electronic Supplementary Material (ESI) for RSC Advances.
This journal is © The Royal Society of Chemistry 2021

Supplementary Information

3D printed silicon-few layer graphene anode for advanced Li-ion batteries

Hossein Beydaghi,[1] Sara Abouali,[1,2] Sanjay B. Thorat,[1,2] Antonio Esau Del Rio Castillo,[1,2] Sebastiano Bellani,[1,2] Simone Lauciello,[1] Silvia Gentiluomo,[1] Vittorio Pellegrini,[1,2*] and Francesco Bonaccorso[1,2*]

[1] Graphene Labs, Istituto Italiano di Tecnologia, via Morego 30, 16163 Genoa, Italy

[2] BeDimensional SpA, Lungotorrente Secca, 30R, 16163 Genoa, Italy

S 1. Morphological characterization of wet-jet milled (WJM)-exfoliated graphene flakes

The morphology of the WJM-produced graphene flakes was characterized by atomic force microscopy (AFM) and transmission electron microscopy (TEM) measurements (**Figure S1**). An AFM image of a representative WJM-produced graphene flake is shown in **Figure S1a**, together with its height profile corresponding to a thickness of ~4.6 nm. The statistical AFM analysis of the thickness of the as-produced flakes (**Figure S1b**) indicates that the data follow a lognormal distribution peaked at ∼2.08 nm. Therefore, the WJM-exfoliated graphene flakes mainly consists of few-layer graphene (sample therefore named WJM-FLG). **Figure S1c** shows a TEM image of the WJM-FLG flakes, indicating that the flakes have a laminar structure with well defined borders. The statistical TEM analysis of the lateral size of the flakes (**Figure S1d**) shows also a lognormal distribution, with a mode at ~1080 nm.

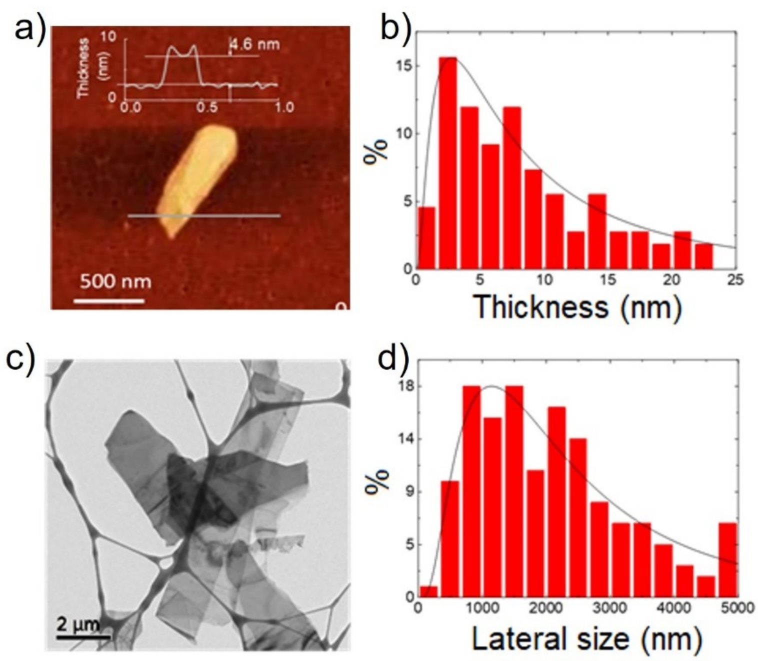

Figure S1. a) AFM image of a representative WJM-FLG flakes and b) the statistical analysis of their thickness. c) TEM image of representative WJM-FLG flakes and d) their later size statistical analysis.

S 2. X-ray photoelectron spectroscopy (XPS) analysis of the WJM-FLG flakes

The chemical properties of the WJM-FLG flakes were evaluated through XPS measurements. **Figure S2a** reports the XPS wide scan, where the C 1s peak is predominant, indicating the absence of other elements. Besides, the high resolution of C 1s and O 1s spectra of the WJM-FLG flakes are also shown in **Figure S2b** and **c**, showing a main peaks at 284.4 eV corresponding to C═C bonds (sp^2 carbon), together with corresponding peak associated to the π–π* interactions at 290.8 eV.[1–3] A second component, centred at 284.8 eV, refers to the C-C bonds (sp^3).[3,4] This peak is associated to the defective edges of the flakes, as well as to environmental contaminations, *i.e.*, adventitious carbon.[5] The other observed peaks at 286.2, and 287.6 eV are attributed to ether/epoxy (C–O–C) and ketone/aldehyde (C═O) functional groups, respectively.[6,7] These peaks are ascribed to the residual solvent (N-Methyl-2-pyrrolidone molecules) that was used during the WJM exfoliation process.[6,7] The presence of minimal oxygen groups is corroborated by the XPS O 1s spectrum of the WJM-FLG flakes (**Figure S2c**).[8,9] These data evidence the bands associated to C-O and O-H, and COO, peaking at 532.8 and 531.8 eV, respectively.[8,10,11] Overall, these results confirm that high-quality WJM-FLG nanoflakes with a negligible contribution of C state besides sp^2 were effectively produced by WJM technique, in agreement with our previous studies.[12–14]

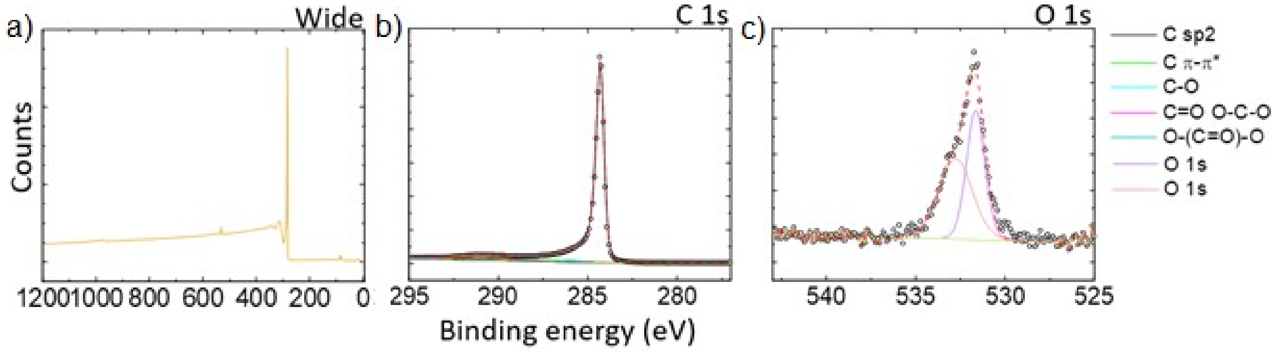

Figure S2. a) XPS wide scan spectrum and b) high-resolution of C 1s and c) O 1s spectra of the WJM-FLG flakes.

S 3. Raman spectroscopy analysis of the material powders

Raman spectroscopy analysis was performed to investigate the structural quality of the material powders (*i.e.*, PLA, doped PPy, WJM-FLG and Si) investigated in the main text (**Figure S3**). The Raman spectrum of the doped PPy exhibits two bands at ~ 1330 and 1590 cm^{-1}, which correspond to the ring-stretching mode of PPy [15] and double bond backbone stretching vibration of PPy [16] bonds, respectively. In the PLA Raman spectrum, the intense peak at ~ 2946 cm^{-1} is assigned to the CH_3 symmetric stretch in the PLA chains.[17] [18] The CH_2 symmetric stretching vibration of PLA appears at 2880 cm^{-1}.[17] The Raman spectrum of WJM-FLG is composed by several characteristic peaks. The G peak, positioned at ∼1585 cm^{-1}, which corresponds to the E_{2g} phonon at the Brillouin zone centre.[19,20] The D peak, which is due to the breathing modes of the sp^2 hybridized carbon rings requiring a breaking on the carbon-ring symmetry for its activation by double resonance. Double resonance also happens as an intra-valley process, *i.e.*, connecting two points belonging to the same cone around K or K',[19] resulting in the rise of the D' peak. The 2D peak (a second order resonance of the D band) centred at ∼2680 cm^{-1} for an excitation wavelength of 514.5 nm in case of a single layer graphene.[19] For few and multi-layer graphene the 2D peak is a superposition of multiple components, the main being the $2D_1$ and $2D_2$ components.[21] The 2D peak is always present, since no defects are required for the activation of two phonons with the same momentum, one being backscatter from the other.[19] In graphite the intensity of the $2D_2$ band is roughly twice the $2D_1$ band [19], while for mechanically exfoliated single layer graphene (SLG) the 2D band is a single and sharp peak, which is roughly 4 times more intense than the G peak.[21] In this regard, the quality of the exfoliated material, in terms of crystalline integrity, is analysed by Raman spectroscopy. Taking into account the intensity ratios of the $2D_1$ and $2D_2$ bands, it is possible to estimate the flake thickness.[22,23]

The intensity variations of the D and D' bands are related to an increase of edges or in-plane defects.[24–26] The defects associated to the peak D are ascribed to the edges of the graphene flakes When the plot of I(D)/I(G) *vs*. FWHM(G) does not show a linear correlation (*e.g.*, $R^2 < 0.6$), the defects are attrbitued to the edges of the graphene flakes.[27,28] Contrarily, I(D)/I(G) *vs*. FWHM(G) shows a linear correlation when the defects occur in the basal planes of the graphene flakes, as occurring for graphene derivatives.[27,28]

As shown in **Figure S3b**, the plot of I(D)/I(G) *vs.* FWHM(G) for WJM-FLG flakes does not show any linear correlation, indicating the absence of structural defect in the graphene basal planes. This

means that the WJM exfoliation does not alter the sp^2 structure of the starting graphite layers. The normalised intensity ratios $I(2D_1)/I(G)$ *vs.* $I(2D_2)/I(G)$ give an insight on the flake thickness (see **Figure S3 c**). As mentioned above, for graphite, the intensity of $2D_2$ peak $[I(2D_2)]$ is roughly double compared to the intensity of $2D_1$ peak $[I(2D_1)]$.[29] Furthermore, the intensity ratio $[I(2D_2)/I(2D_1)]$ decreases as the flake thickness is reduced,[19] until the 2D band can be fitted by a single Lorentzian, highlighting that the flakes are electronically decoupled.[30] The dashed line in **Figure S3 c** represents the multilayer condition (~5 layers) [21,31] $[I(2D_1)/I(G) = I(2D_2)/I(G)]$ separating the data set, while the points below the line $[I(2D_1)/I(G) < I(2D_2)/I(G)]$ are considered graphitic flakes, and the points above the line $[I(2D_1)/I(G) > I(2D_2)/I(G)]$ are considered FLG and SLG.[19,21,30] Lastly, the Raman spectrum of the F5 sample resembles the one of doped PPy. Since the doped PPy cover the WJM-FLG flakes, the D, G and 2D bands associated to the WJM-FLG flakes are masked by the signal of the doped PPy.

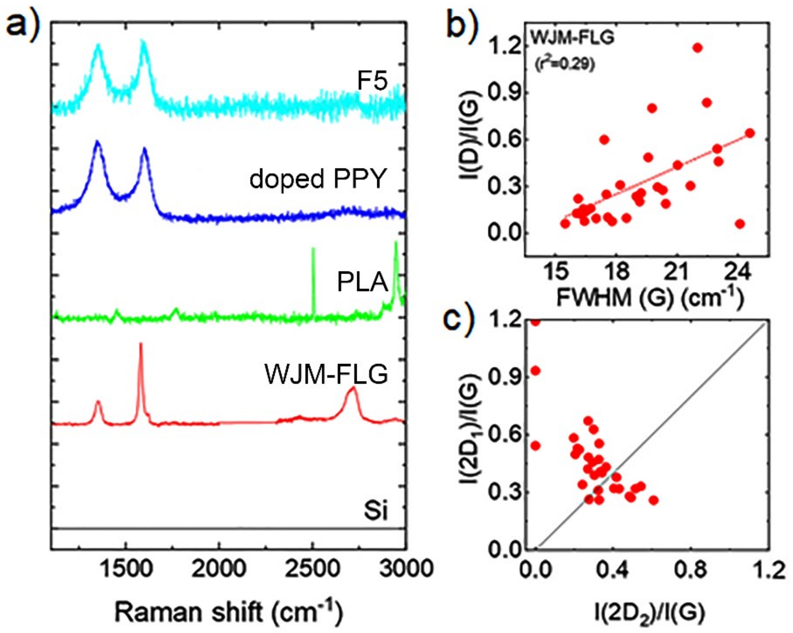

Figure S3. a) Comparison between the Raman spectra of the various powder b) Statistical analysis of FWHM(G) *vs.* I(D)/I(G) and their linear correlation (dashed line) and c) The normalised integral intensities of the peaks $2D_1$ and $2D_2$ showing the distribution of FLG and graphite. The dashed line represents the condition where $I(2D_1)/I(G) = I(2D_2)/I(G)$.

S 4. X-ray diffraction (XRD) spectroscopy analysis of the material powders

Figure S4 shows the XRD patterns of Si and F5 electrode, whose composition is described in the main text (**Table 1**). The corresponding results were discussed in the main text.

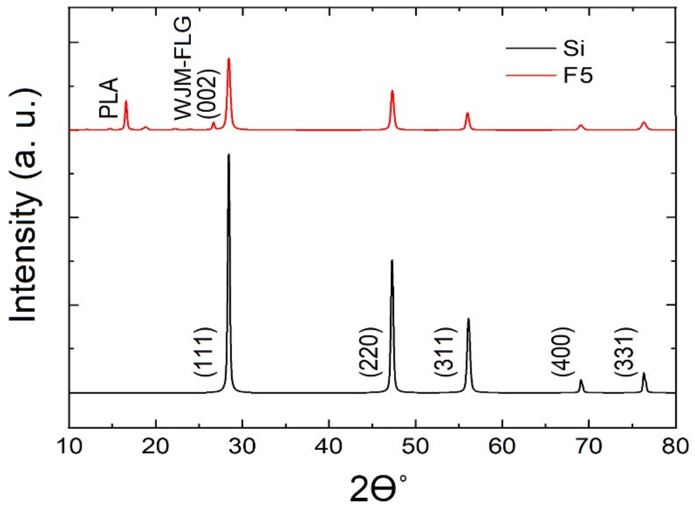

Figure S4. a) XRD patterns of pristine Si and F5 electrode.

S5. Mechanical and thermal properties of filaments

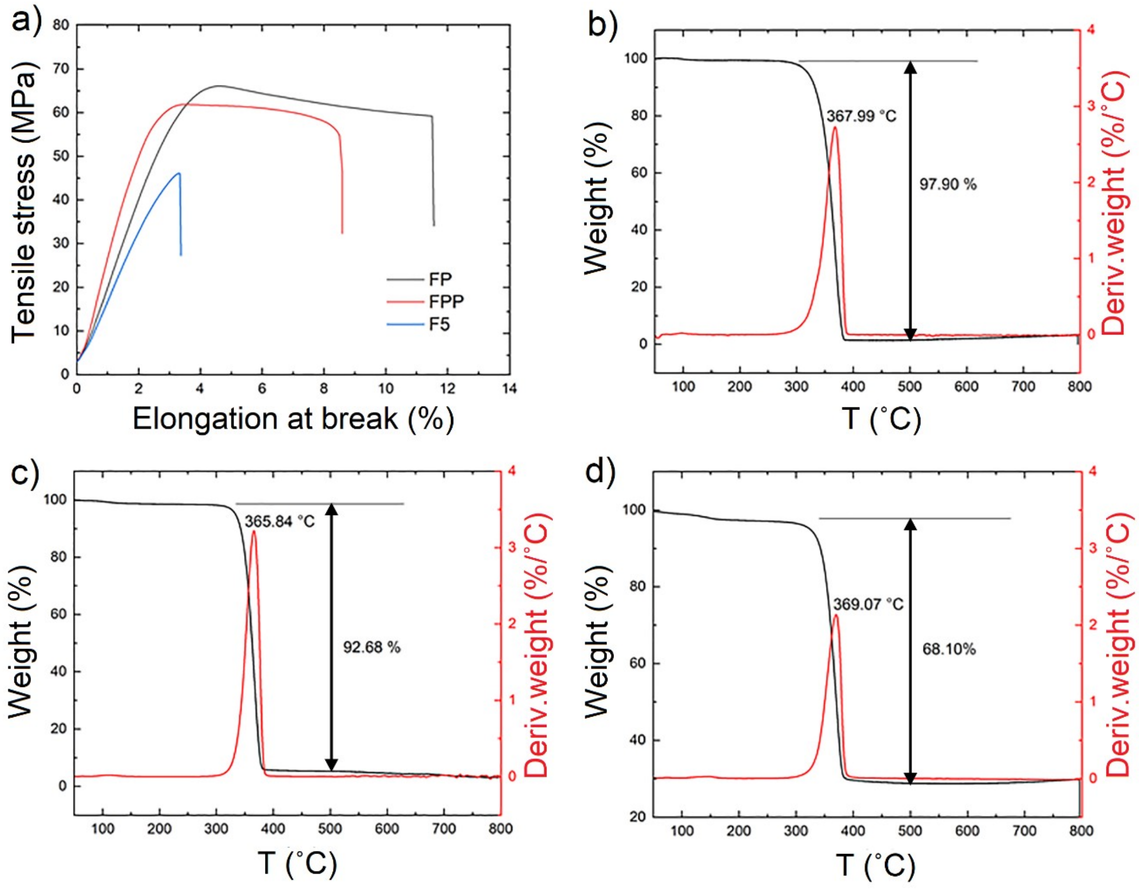

Figure S5. a) Stress-strain curves and b,c,d) TGA curves of the pristine PLA (FP) and doped PPy/PLA composite (FPP), and F5 filaments, respectively. The composition of the filaments is described in Table 1 of the main text. T (˚C)

The mechanical properties of the filaments are key characteristics for 3D printability of filaments that need to be investigated in the fabrication and development of 3D FDM-printable products. **Figure S5a** shows the stress-strain curve measured for the F5 filament, in comparison to those measured for the filaments based on only pristine PLA (FP) and doped PPy/PLA composite (FPP). Being the PLA mechanically stronger than PPy,[32] the FPP filament shows a tensile strength of 62.14 MPa, which is lower than the one of FP filament (66.03 MPa). Furthermore, the addition of Si nanoparticles and WJM-FLG flakes into the PLA/doped PPy composite increases the brittleness of the filament, as a consequence of the decrease of the mobility of the polymer chains when the WJM-FLG flakes and Si nanoparticles are incorporated within the polymeric layers.[33–35] However, the elongation at break (E_b) and the tensile strength of the electrode F5 are still as high as 3.1% and 45.58 MPa, respectively. These values are adequate for printing filaments having high

electrical conductivity (*i.e.*, 5.19 S cm^{-1}). By increasing the Si content above 29.5 wt% (as used in F5), the filaments start to loose their malleability and mechanical strength, which are required to follow the pipe connected with the 3D printer nozzle and to resist the mechanical stresses caused by the printing process.[36]

The TGA analysis was performed on the produced filaments to study the thermal stability in the 50 - 800 °C temperature range (**Figure S5b to d**), while confirming the exact content of materials in the printed filaments. The weight loss of the filaments begins at *ca.* 300 °C, with a fast decay up to 400 °C, which is reflected in the weight derivative curves, with peaks between 365-370 °C. The observed weight loss is attributed to the degradation of the polymer chains.[32] **Figure S5b,c** show that almost 98% of the PLA weight and 93% of the PLA/doped PPy weight are lost between 300 and 400 °C. Therefore, only 7% of the PLA and doped-PPy weights remains in the F5 filament. Contrary to the polymers, Si nanoparticles are stable at temperature up to 800 °C, and weight loss is not observed in their TGA curve.[37,38] Above 800 °C, the F5 material looses 68 % of its weight. Since the Si/WJM-FLG amount in the F5 filament is 35 wt%, the discrepancy with residuals wt% calculated by TGA (32 wt%) is ascribed to the weight loss associated to the WJM-FLG in the Si/WJM-FLG composite, in agreement with previous literature (~ 20 wt% weight loss of Si/graphene composites).[39,40]

S6. Flexibility and printability of filament

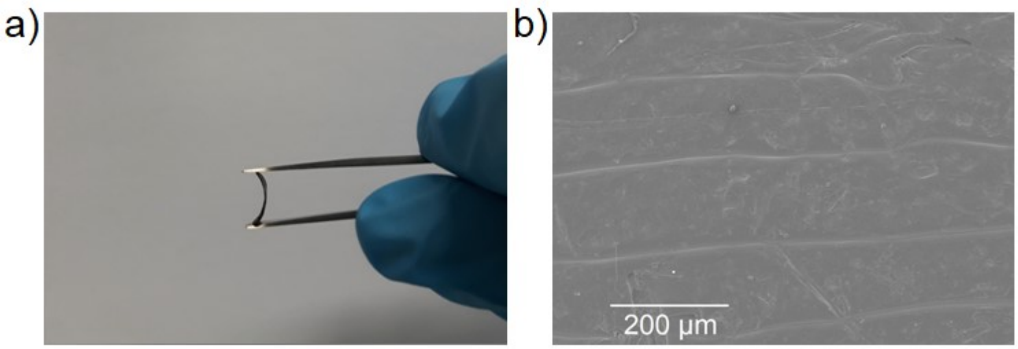

Figure S6. a) Photograph of the 3D printed F5 electrode, which was bent to show its flexibility. b) SEM image of the 3D printed F5 electrode.

The mechanical flexibility of the electrode, when assembled in a Li-ion battery, plays a major role in maintaining its cyclic stability during lithiation/de-lithiation processes.[41] **Figure S6a** shows a photograph of a 3D printed F5 electrode, which was bent to an angle of 120° to prove its flexibility. **Figure S6b** shows the top-view scanning electron microscopy (SEM) image of the same F5 electrode, evidencing a structure composed by consecutively printed filaments.

S7. Cyclic performance of the 3D printed anodes

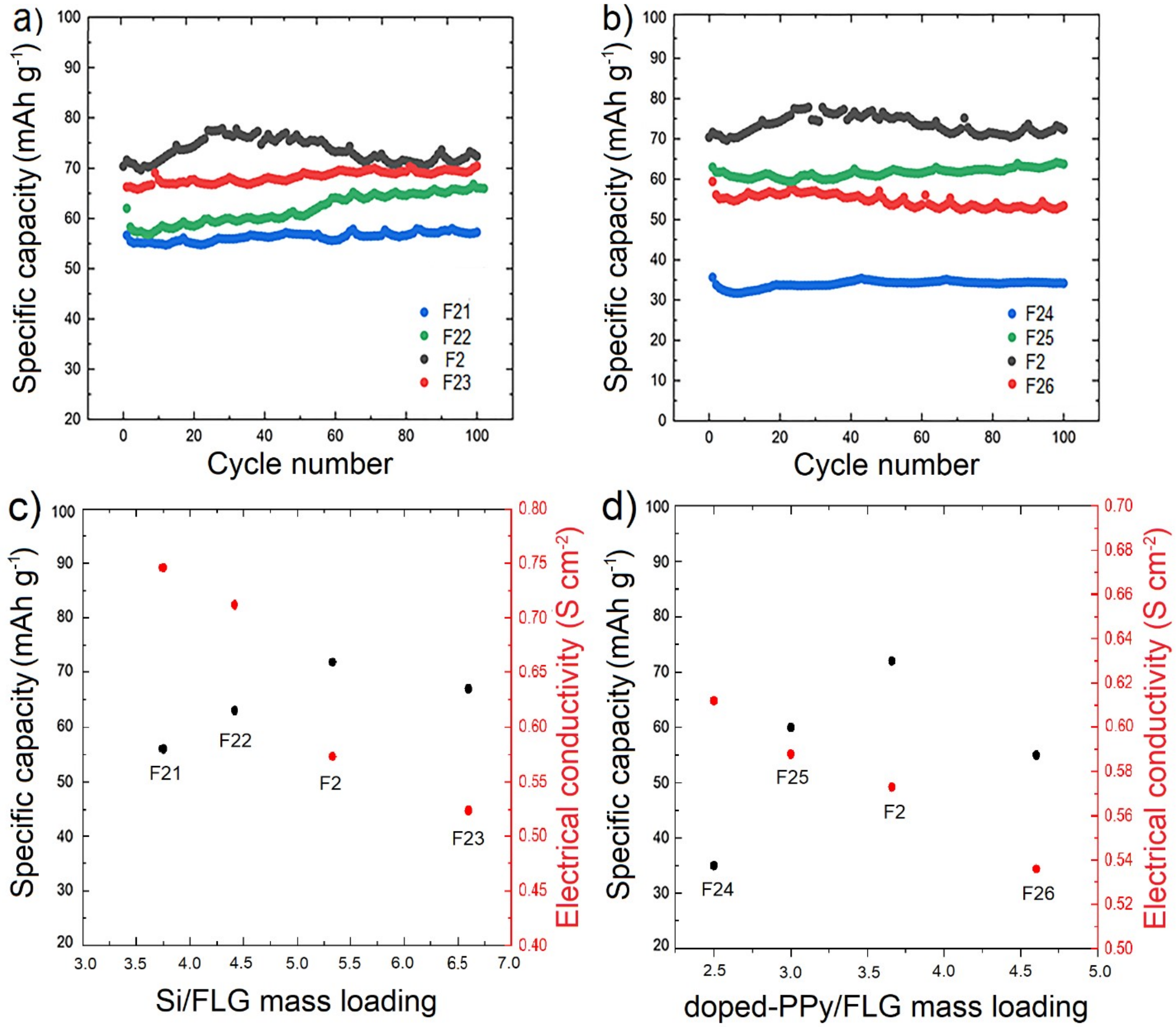

Figure S7. Cyclic performance of the 3D printed anodes in the half-cell at the current density of 20 mA g^{-1} with a) constant amount of PLA and carbon black doped PPy (70 and 11 wt%, respectively) and the different weight ratio of Si:WJM-FLG nanoflakes, b) constant amount of PLA and Si (70 and 16 wt%, respectively) and the different weight ratio of carbon black-doped PPy:WJM-FLG. Dependence of the specific capacity and the conductivity on c) the Si:WJM-FLG mass ratio and d) the doped-PPy:WJM-FLG mass ratio.

S8. High-resolution SEM image of 3D printed electrode

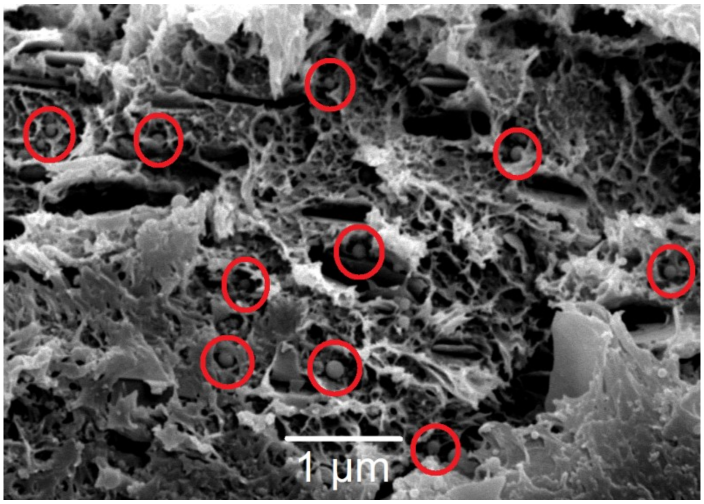

Figure S8. Cross-sectional high-resolution SEM image of the F5 electrode after 350 galvanostatic charge/discharge cycles at the current density of 20 mA g^{-1}. The red circles indicate the Si nanoparticles, which are encapsulated by surrounding carbonaceous materials composing the electrode.

S9. Comparison of our 3D printed electrode performance with literature

Table S1. Comparison between gravimetric and volumetric capacities of the F5 electrode after 100 cycles with those of electrodes produced by FDM 3D printing reported in literature

Electrodes	Gravimetric capacity (mAh g^{-1})	Volumetric capacity (mAh cm^{-3})	Current density (mA g^{-1})	Ref.
F5	343	58.6	20	This Work
PLA/graphite	200	-	18.6	36
PLA/graphene/LTO[a]	-	3.5	20	42
PLA/graphene	35	-	10	43
PLA/graphene	150	-	40	44

[a]; Lithium titanate

References

1 A. Siokou, F. Ravani, S. Karakalos, O. Frank, M. Kalbac and C. Galiotis, *Appl. Surf. Sci.*, 2011, **257**, 9785–9790.

2 S. Stankovich, D. A. Dikin, R. D. Piner, K. A. Kohlhaas, A. Kleinhammes, Y. Jia, Y. Wu, S. T. Nguyen and R. S. Ruoff, *Carbon N. Y.*, 2007, **45**, 1558–1565.

3 J. Díaz, G. Paolicelli, S. Ferrer and F. Comin, *Phys. Rev. B*, 1996, **54**, 8064–8069.

4 J. C. Lascovich, R. Giorgi and S. Scaglione, *Appl. Surf. Sci.*, 1991, **47**, 17–21.

5 G. Salitra, A. Soffer, L. Eliad, Y. Cohen and D. Aurbach, *J. Electrochem. Soc.*, 2000, **147**, 2486–2493.

6 R. Buzio, A. Gerbi, S. Uttiya, C. Bernini, A. E. Del Rio Castillo, F. Palazon, A. S. Siri, V. Pellegrini, L. Pellegrino and F. Bonaccorso, *Nanoscale*, 2017, **9**, 7612–7624.

7 H. Sun, A. E. Del Rio Castillo, S. Monaco, A. Capasso, A. Ansaldo, M. Prato, D. A. Dinh, V. Pellegrini, B. Scrosati, L. Manna and F. Bonaccorso, *J. Mater. Chem. A*, 2016, **4**, 6886–6895.

8 Y. Nanri, T. Wada, H. Yoshitani, H. Fukui, A. Nakasuga, T. Tsumura and M. Toyoda, *FlatChem*, 2020, **24**, 100206.

9 L. Zhou, L. Fox, M. Włodek, L. Islas, A. Slastanova, E. Robles, O. Bikondoa, R. Harniman, N. Fox, M. Cattelan and W. H. Briscoe, *Carbon N. Y.*, 2018, **136**, 255–261.

10 A. V Ramya, A. N. Mohan and B. Manoj, *Mater. Sci.*, 2016, **34**, 330–336.

11 Z. Sun, J. Masa, P. Weide, S. M. Fairclough, A. W. Robertson, P. Ebbinghaus, J. H. Warner, S. C. E. Tsang, M. Muhler and W. Schuhmann, *J. Mater. Chem. A*, 2015, **3**, 15444–15450.

12 A. E. Del Rio Castillo, V. Pellegrini, A. Ansaldo, F. Ricciardella, H. Sun, L. Marasco, J. Buha, Z. Dang, L. Gagliani, E. Lago, N. Curreli, S. Gentiluomo, F. Palazon, M. Prato, R. Oropesa-Nuñez, P. S. Toth, E. Mantero, M. Crugliano, A. Gamucci, A. Tomadin, M. Polini and F. Bonaccorso, *Mater. Horizons*, 2018, **5**, 890–904.

13 S. Bellani, E. Petroni, A. E. Del Rio Castillo, N. Curreli, B. Martín-García, R. Oropesa-Nuñez, M. Prato and F. Bonaccorso, *Adv. Funct. Mater.*, 2019, **29,** 1807659.

14 S. Bellani, B. Martín-García, R. Oropesa-Nuñez, V. Romano, L. Najafi, C. Demirci, M. Prato, A. E. Del Rio Castillo, L. Marasco, E. Mantero, G. D'Angelo and F. Bonaccorso, *Nanoscale Horizons*, 2019, **4**, 1077–1091.

15 Y.-C. Liu and B.-J. Hwang, *Synth. Met.*, 2000, **113**, 203–207.

16 G. Han, J. Yuan, G. Shi and F. Wei, *Thin Solid Films*, 2005, **474**, 64–69.

17 A. Wesełucha-Birczyńska, A. Frączek-Szczypta, E. Długoń, K. Paciorek, A. Bajowska, A. Kościelna and M. Błażewicz, *Vib. Spectrosc.*, 2014, **72**, 50–56.

18 D. Qin and R. T. Kean, *Appl. Spectrosc.*, 1998, **52**, 488–495.

19 A. C. Ferrari and D. M. Basko, *Nat. Nanotechnol.*, 2013, **8**, 235–246.

20 L. Yang, J. Deslippe, C.-H. Park, M. L. Cohen and S. G. Louie, *Phys. Rev. Lett.*, 2009, **103**, 186802.

21 A. C. Ferrari, J. C. Meyer, V. Scardaci, C. Casiraghi, M. Lazzeri, F. Mauri, S. Piscanec, D. Jiang, K. S. Novoselov, S. Roth and A. K. Geim, *Phys. Rev. Lett.*, 2006, **97**, 187401.

22 L. Najafi, S. Bellani, R. Oropesa-Nuñez, B. Martín-García, M. Prato and F. Bonaccorso, *ACS Appl. Energy Mater.*, 2019, **2**, 5373–5379.

23 L. Najafi, R. Oropesa-Nuñez, B. Martín-García, F. Drago, M. Prato, V. Pellegrini, F. Bonaccorso and S. Bellani, *Mater. Adv.*, 2020, **1**, 387–402.

24 M. M. Lucchese, F. Stavale, E. H. M. Ferreira, C. Vilani, M. V. O. Moutinho, R. B. Capaz, C. A. Achete and A. Jorio, *Carbon N. Y.*, 2010, **48**, 1592–1597.

25 A. Eckmann, A. Felten, A. Mishchenko, L. Britnell, R. Krupke, K. S. Novoselov and C. Casiraghi, *Nano Lett.*, 2012, **12**, 3925–3930.

26 A. C. Ferrari, *Solid State Commun.*, 2007, **143**, 47–57.

27 M. V Bracamonte, G. I. Lacconi, S. E. Urreta and L. E. F. Foa Torres, *J. Phys. Chem. C*, 2014, **118**, 15455–15459.

28 J. N. Coleman, *Acc. Chem. Res.*, 2013, **46**, 14–22.

29 M. S. Dresselhaus, G. Dresselhaus and M. Hofmann, *Philos. Trans. R. Soc. A Math. Phys. Eng. Sci.*, 2008, **366**, 231 – 236.

30 J.-B. Wu, M.-L. Lin, X. Cong, H.-N. Liu and P.-H. Tan, *Chem. Soc. Rev.*, 2018, **47**, 1822–1873.

31 A. Das, B. Chakraborty and A. K. Sood, in *Bulletin of Materials Science*, 2008.

32 F. Zhang, Y. Xia, L. Wang, L. Liu, Y. Liu and J. Leng, *ACS Appl. Mater. Interfaces*, 2018, **10**, 35526–35532.

33 H. Beydaghi, A. Bagheri, P. Salarizadeh, S. Kashefi, K. Hooshyari, A. Amoozadeh, T. Shamsi, F. Bonaccorso and V. Pellegrini, *Ind. Eng. Chem. Res.*, 2020, **59,** 6589–6599.

34 H. Beydaghi and M. Javanbakht, *Ind. Eng. Chem. Res.*, 2015, **54,** 7028–7037.

35 H. Beydaghi, L. Najafi, S. Bellani, A. Bagheri, B. Martín-García, P. Salarizadeh, K. Hooshyari, S. Naderizadeh, M. Serri, L. Pasquale, B. Wu, R. Oropesa-Nuñez, Z. Sofer, V. Pellegrini and F. Bonaccorso, *J. Mater. Chem. A*, 2021, **9**, 6368–6381.

36 A. Maurel, M. Courty, B. Fleutot, H. Tortajada, K. Prashantha, M. Armand, S. Grugeon, S. Panier and L. Dupont, *Chem. Mater.*, 2018, **30**, 7484–7493.

37 C. Li, C. Liu, K. Ahmed, Z. Mutlu, Y. Yan, I. Lee, M. Ozkan and C. S. Ozkan, *RSC Adv.*, 2017, **7**,

36541–36549.

38 W. J. Lee, T. H. Hwang, J. O. Hwang, H. W. Kim, J. Lim, H. Y. Jeong, J. Shim, T. H. Han, J. Y. Kim, J. W. Choi and S. O. Kim, *Energy Environ. Sci.*, 2014, **7**, 621–626.

39 X. Ma, G. Hou, Q. Ai, L. Zhang, P. Si, J. Feng and L. Ci, *Sci. Rep.*, 2017, **7**, 9642.

40 H. Liu, Z. Shen, S. Liang, L. Liu, M. Yi, X. Zhang and S. Ma, *New J. Chem.*, 2016, **40**, 7053–7060.

41 R. Malik, Q. Huang, L. Silvestri, D. Liu, V. Pellegrini, L. Marasco, E. Venezia, S. Abouali, F. Bonaccorso, M. J. Lain, D. Greenwood, G. West, P. R. Shearing and M. J. Loveridge, 2D Mater. 2021, **8,** 015012.

42 C. Reyes, R. Somogyi, S. Niu, M. A. Cruz, F. Yang, M. J. Catenacci, C. P. Rhodes and B. J. Wiley, *ACS Appl. Energy Mater.*, 2018, **1**, 5268–5279.

43 C. W. Foster, M. P. Down, Y. Zhang, X. Ji, S. J. Rowley-Neale, G. C. Smith, P. J. Kelly and C. E. Banks, *Sci. Rep.*, 2017, **7**, 42233.

44 C. W. Foster, G.-Q. Zou, Y. Jiang, M. P. Down, C. M. Liauw, A. Garcia-Miranda Ferrari, X. Ji, G. C. Smith, P. J. Kelly and C. E. Banks, *Batter. Supercaps*, 2019, **2**, 448–453.
